# Pathogenic modulators of cellular senescence

**DOI:** 10.18699/vjgb-26-72

**Published:** 2026-09

**Authors:** E.V. Simoroz, Y.V. Antonov, G.S. Muravyov, J. Vasilevska, E.I. Rogaev

**Affiliations:** Sirius University of Science and Technology, Sirius Federal Territory, Krasnodar region, Russia; Sirius University of Science and Technology, Sirius Federal Territory, Krasnodar region, Russia; Sirius University of Science and Technology, Sirius Federal Territory, Krasnodar region, Russia; Sirius University of Science and Technology, Sirius Federal Territory, Krasnodar region, Russia; UMass Chan Medical School, Department of Psychiatry, Shrewsbury, United States

**Keywords:** senescence, viruses, bacteria, pathogenic modulators, старение, вирусы, бактерии, патогенные модуляторы

## Abstract

Cellular senescence is characterized by irreversible cell-cycle arrest, with cells remaining viable and metabolically active. This state features a proinflammatory senescence-associated secretory phenotype (SASP) that can harm neighboring tissues. Accumulation of senescent cells accelerates age-related physiological decline and associated pathologies. Furthermore, cellular senescence is implicated in various physiological processes, including embryonic development, wound healing, tumor progression, and immune response regulation. The complexity of the aging process arises from its diverse underlying mechanisms, potential reversibility, and intrinsic heterogeneity. Experimental gerontology focuses on identifying pathogenic modulators that regulate the formation and accumulation of senescent cells, as well as investigating their impact on tissue function. Within the field of experimental gerontology, considerable attention is devoted to identifying pathogenic modulators that regulate the formation and accumulation of senescent cells, as well as to investigating their impact on tissue function. Of particular interest is the characterization of novel molecular mechanisms that govern cellular aging. Key pathogenic molecular pathways include the p53-dependent senescence pathway, the p16INK4a/pRb pathway, and non-canonical pathways like IFIH1-MAVS, which contribute to oxidative stress, DNA damage, activation of SASP, and other cellular dysfunction. This study critically examines how viral and bacterial agents induce cellular senescence, particularly in vitro, reviewing the regulatory mechanisms involved. It discusses genetic variants affecting infection susceptibility and categorizes senescence markers. Investigating the molecular mechanisms underlying cellular aging presents promising avenues for the development of targeted and effective therapeutic interventions. Such strategies may include the selective induction of senescence in cancer cells, suppression of senescence to mitigate age-related diseases, or the comprehensive modulation of aging processes to optimize clinical outcomes.

## Abstract (ru)

Клеточное старение (сенесценция) представляет собой необратимую утрату способности к пролиферации при сохранении жизнеспособности и метаболической активности клетки. Этот процесс сопровождается формированием провоспалительного секреторного фенотипа, ассоциированного со старением (SASP), который способен оказывать повреждающее воздействие на окружающие ткани и организм в целом. Накопление сенесцентных клеток в тканях способствует ускорению возрастных изменений организма и развитию сопутствующих патологий, а также оказывает влияние на различные физиологические процессы, включая эмбриональное развитие, заживление ран, рост опухолей и регуляцию иммунного ответа. Сложность процесса старения обусловлена многообразием его механизмов, потенциальной обратимостью и гетерогенностью. В области экспериментальной геронтологии особое внимание уделяется выявлению патогенных модуляторов, регулирующих образование и накопление сенесцентных клеток, и исследованию их влияния на функциональное состояние тканей. Особый интерес представляет выявление новых молекулярных механизмов, участвующих в регуляции процессов клеточного старения. Основные патогенно опосредованные молекулярные пути включают p53-опосредованное старение и путь p16INK4a/pRb, а также неканонические механизмы, такие как IFIH1-MAVS, которые вызывают окислительный стресс, повреждение ДНК, активацию SASP и другие клеточные нарушения. В данной работе проводится анализ влияния вирусов и бактерий на процессы клеточного старения с основным акцентом на индукцию сенесценции in vitro. Представлен всесторонний обзор регуляторных механизмов, посредством которых бактерии и вирусы инициируют старение клеток. Описаны генетические варианты, которые обусловливают предрасположенность к инфекциям, что, в свою очередь, влияет на патоген-индуцируемые процессы старения. Кроме того, систематизированы и проанализированы маркеры, применяемые для идентификации сенесцентных клеток. Исследование молекулярных механизмов клеточного старения открывает возможности для создания целенаправленных и эффективных терапий. Эти стратегии могут избирательно активировать старение в раковых клетках, подавлять его для замедления возрастных заболеваний или комплексно регулировать процессы старения для оптимальных результатов.

## Introduction

Infectious agents are factors that contribute to the aging process, exerting their effects through two interrelated mechanisms. First, they cause direct damage to cells and tissues through infection, leading to various age-related pathologies, including cardiovascular diseases (notably stroke) (Donato et al., 2018; Smolgovsky et al., 2021), neurodegenerative disorders (Alzheimer’s disease and Parkinson’s disease) (Pringsheim et al., 2014; Trevisan et al., 2019; Blackhurst, Funk, 2023), immune system dysfunctions (Sadighi Akha, 2018), as well as oncological diseases (DeSantis et al., 2019; Kannampuzha et al., 2023). Second, they indirectly promote the induction of aging and the accumulation of senescent cells, which, in turn, is recognized as one of the key mechanisms underlying the development of chronic diseases and overall decline in organismal viability. These changes lead to a progressive decline in physical and cognitive functions, as well as to an increased risk of disease and mortality (Aman et al., 2021).

Aging in mammals, including humans, is associated with progressive dysfunction of the immune system, termed “immunosenescence” (Arakelyan et al., 2025). This process is characterized by a quantitative decline and reduced functional activity of key immune cell populations, as well as disruption of immune homeostasis. Consequently, it leads to a weakened adaptive immune response, increasing susceptibility to infectious diseases and exacerbating their severity in old age. Immunosenescence is also considered one of the drivers of systemic inflammation and the acceleration of aging processes. Epidemiological data serve as clinical evidence of agerelated vulnerability, demonstrating a higher incidence of SARS-CoV-2 infection and a greater risk of complications among elderly individuals compared to the 18–39 age cohort. In contrast, the immune response in childhood is characterized by high activity and adaptive potential, reflecting pronounced age-related dynamics in the immune system’s response to pathogens (McGovern et al., 2023; Schmitt et al., 2023).

Cellular senescence is categorized into replicative and stress-induced senescence. Replicative senescence is associated with telomere shortening and the activation of pathways such as p53 and p21. Stress-induced senescence is driven by increased expression of the CDKN2A gene and the p16 protein, leading to cell cycle arrest (Yan et al., 2024).

For reliable identification of senescent cells, a combination of markers is used (Fig. 1): for example, β-galactosidase activity (SA-β-gal) and the presence of the senescence-associated secretory phenotype (SASP) (Fafián- Labora, O’Loghlen, 2020; Ogrodnik et al., 2024; Wyles et al., 2024).

**Fig. 1.**
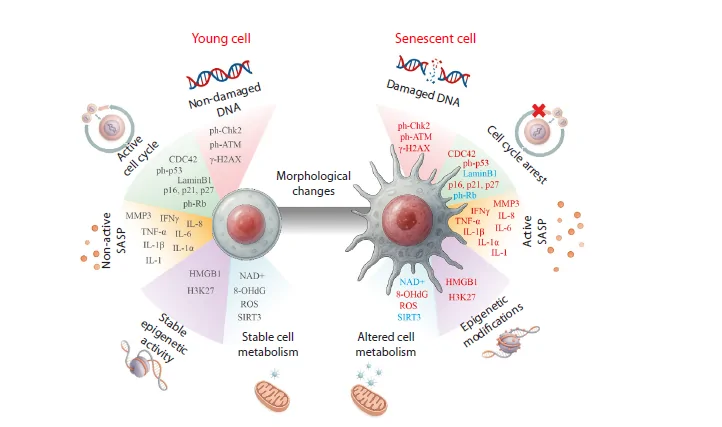
Schematic representation comparing a young and a senescent cell, indicating the main changes and classical markers. Markers, the expression of which is upregulated in senescent cells, are highlighted in red compared to the gray control, whereas markers with downregulated expression in senescent cells are indicated in blue.

There is evidence indicating that the senescent state represents a phenomenon with a high, yet not absolute, barrier to reversibility. A clear illustration of this is the induction of Yamanaka factors (Oct4, Sox2, Klf4, c-Myc), which can reprogram the cell and eliminate epigenetic markers of aging, reverting even senescent cells to a pluripotent state, with the subsequent possibility of their differentiation into various cell types. Under experimental conditions, reversal of senescence can be induced through genetic or epigenetic manipulations; however, this approach carries substantial risks, primarily the potential for oncogenesis (Aguirre et al., 2023; Jiang et al., 2026). Another example is provided by extracellular vesicles derived from embryonic stem cells. These vesicles have been shown to act as mediators modulating the activity of neighboring cells: they enhanced proliferative potential and reduced the expression of SA-β-gal as well as other markers of aging, such as mitochondrial membrane potential and reactive oxygen species levels. Furthermore, a decrease in the expression of classical markers of cellular senescence – p21WAF1/CIP1 (p21) and p16INK4a (p16) – was observed. This process has been found to be mediated by inhibition of the p38 MAPK signaling pathway (Liu Yu. et al., 2023).

The study by M. Reimann and colleagues (2024) demonstrates that the degree of reversibility of senescent states is more complex and context-dependent. Following exposure to a pathogen or a stressor, the development of the senescent phenotype can occur rapidly – within days or weeks. In the early stages, cells may retain a reversible state and the ability to restore function. However, as damage accumulates and corresponding signaling pathways (e. g. those mediated by p53 and p16INK4a) become activated, senescence stabilizes and acquires an irreversible character (Reimann et al., 2024).

In addition to age-related susceptibility to infections, it is important to note that individual aging trajectories can vary significantly depending on genetics. Genetic mutations may determine individual aging trajectories in response to infectious challenges. Such genes typically include those that maintain immune homeostasis (e. g. PTPN2 and FOXO3) (Hale et al., 2020; Jeanpierre et al., 2024), as well as genes responsible for adaptive immunity, including HLA genes, the Toll-like receptor (TLR) family, and interferon regulatory factors (IRF) (Zhang et al., 2015; De Jong et al., 2018; Augusto et al., 2023). Furthermore, genes encoding receptors used by pathogens for cell entry also play a significant role.

It should be noted that research on aging processes in the context of infectious diseases is a rapidly evolving field of science, with a substantial number of new studies published monthly (Alam et al., 2021; Ryu et al., 2021; Masters et al., 2022; Aguado et al., 2023; Gu et al., 2025). This work reflects growing interest in this topic and opens prospects for identifying novel targets in combating infections and associated pathologies. The present review examines aging as a direct consequence of pathogen exposure, with a particular focus on in vitro studies that describe the activated signaling pathways and the identified markers of this process.

## Bacteria as positive modulators of aging

There is growing interest in studying the role of the microbiome and bacterial toxins in regulating the aging processes of the host organism. Bacteria can either promote or delay cellular aging processes, depending on their species and metabolic activity. Beneficial probiotic microorganisms synthesize metabolites, such as short-chain fatty acids, which possess anti-inflammatory and antioxidant effects. These metabolites contribute to slowing down the aging processes by strengthening immunity and reducing levels of chronic inflammation.

In the study by S.-J. Han and colleagues (2023), it was demonstrated that different microbiota profiles shape distinct levels of immune optimization, which is particularly important in the context of combating pathogens and inflammation. Interventions aimed at modulating the microbiota, such as the use of probiotics, may represent effective strategies for preventing or delaying pathogeninduced aging. An optimal microbiome balance enhances immune responses, reduces inflammatory processes, and decreases the risk of developing age-related diseases (Han et al., 2023)

However, some pathogens, upon infecting cells, are capable of manipulating molecular pathways, which often leads to premature aging. This effect may manifest through DNA damage in host cells or the secretion of genotoxic proteins, thereby exacerbating cellular aging processes and negatively affecting the overall state of the organism (Chumduri et al., 2016). For instance, the bacterium Helicobacter pylori, which causes chronic atrophic gastritis, acts as a factor inducing cellular senescence and contributing to cancer development, representing an evident phenomenon in the gastric mucosa. It has been demonstrated that this bacterium causes dysregulation of the Erk signaling pathway, leading to the accumulation of the cyclin-dependent kinase inhibitor p21Cip1/Waf1, which results in the arrest of cell proliferation characteristic of the aging process (Saito et al., 2010). Another study showed that H. pylori induces senescence of gastric epithelial cells both in vitro and in vivo through activation of the chemokine receptor CXCR2 signaling pathway (Cai et al., 2021).

Another example is provided by the bacteria Porphyromonas gingivalis and P. asaccharolytica, identified in significant quantities in patients with colorectal cancer. Cocultivation of human fibroblasts in a medium containing culture supernatants of these bacteria led to irreversible cell cycle arrest, accompanied by increased expression of p16INK4a and p21Cip1/Waf1, as well as SASP factors (IL-1 and IL-6). Similar results were obtained in intestinal epithelial organoids. This effect is attributed to the high secretion of butyrate – a short-chain fatty acid – by these bacteria. The authors hypothesize that the impact of bacteria in inducing senescence processes contributes to the development and progression of colorectal cancer (Okumura et al., 2021).

Disorders of the hematopoietic system also represent a prominent sign of aging, accompanied by a reduced capacity for self-renewal, an increased number of myeloid cells, accumulation of DNA damage, and epigenetic and transcriptional changes. It has been suggested that the microbiota also plays a significant role in these processes. The study by L.V. Kovtonyuk et al. (2022) showed that in aged mice, the bone marrow releases more IL-1α and IL-1β, and patterns associated with microbes, such as TLR4 and TLR8 ligands, are detected in the blood. The authors suggest that the microbiome stimulates bone marrow cells to increase cytokine production. Supporting this hypothesis, it was found that bone marrow cells from aged mice, upon lipopolysaccharide stimulation, exhibit a more pronounced and sustained IL-1α/β production response both in vitro and in vivo. Moreover, antibiotic treatment reduced IL-1 levels in the bone marrow, confirming the role of the microbiome in shaping age-related changes in hematopoiesis via the IL-1R1 pathway (Kovtonyuk et al., 2022).

Similar to what has been described above, aging of the cardiovascular system, driven by endothelial cell senescence, may also be a consequence of bacterial activity. It has been shown that the bacterium Clostridium sp. ASF356, characterized by high production of the bacterial metabolite phenylacetic acid and its by-product phenylacetylglutamine, is significantly more abundant in aged mice and humans. In young mice, infection with these bacteria increases blood levels of phenylacetic acid and induces endothelial cell senescence, increasing mitochondrial H2O2. In aortic cells of infected mice, increased expression of the senescence marker p16INK4a, DNA damage (γ-H2A.X), elevated SASP factors, as well as increased β-galactosidase activity, are observed (Saeedi Saravi et al., 2025).

Another significant factor capable of inducing aging processes is Mycobacterium tuberculosis, the causative agent of tuberculosis. Infected patients exhibit accelerated epigenetic aging by approximately 12.7 years relative to their chronological age, accompanied by elevated levels of SASP components CXCL9, CXCL10, and TNFα (Bobak et al., 2022). Studies on murine alveolar-like macrophages have demonstrated that M. tuberculosis induces accelerated senescence in vitro. This results in reduced cell proliferation, morphological changes, activation of DNA damage pathways, and increased expression of senescence markers such as p21 and SA-β-galactosidase. Senescent macrophages secrete pro-inflammatory cytokines (SASP) and promote senescence of neighboring cells (Scarpa et al., 2024). It is known that M. tuberculosis infects approximately one-third of the world’s population and causes about two million deaths annually. Nevertheless, only 10 % of infected individuals develop clinically manifest tuberculosis (Lin, Flynn, 2010). This phenomenon is likely due to genetic predisposition. A significant association of the disease with a single nucleotide polymorphism (SNP) in exon rs4331426, located on chromosomal region 18q11.2, has now been established (Thye et al., 2010). However, functional confirmation of this association is lacking.

Similar to the causative agent of tuberculosis, the malaria parasite Plasmodium falciparum is capable of inducing accelerated cellular senescence, where host genetic factors play a key role in the predisposition to developing severe malaria and its complications. Analysis of postmortem brain and peripheral blood samples from malaria patients revealed elevated levels of the p21 marker in the absence of changes in p16 expression (Hellani et al., 2024). Furthermore, this pathogen contributes to accelerated aging of erythrocytes, primarily due to alterations in the biochemical composition of the membrane, manifested by reduced phospholipid and cholesterol content. These changes play a significant role in the pathogenesis of malaria. The authors hypothesize that these alterations may result from peroxidative oxidative damage induced by the parasites (Omodeo-Salè et al., 2003). In a genome-wide association study (GWAS) comparing patients and healthy individuals, the most significant association signal was detected near the beta-hemoglobin gene, which contains the classic sickle hemoglobin S (HbS) polymorphism. It should be noted that homozygotes for the rs334 allele develop lifethreatening sickle cell anemia, whereas heterozygotes for this variant have an approximately tenfold reduced risk of severe malaria. Additionally, further genetic variants requiring additional study and validation have been identified, including mutations in the SCO1 and DDC genes (Jallow et al., 2009).One of the pressing issues in contemporary immunology is the study of immunosenescence induced by various bacterial agents. The study by S.L. Mathiasen and colleagues (2021) demonstrated that genotoxins from pathogenic Gram-negative bacteria (e. g., Escherichia coli, Shigella dysenteriae, Campylobacter jejuni, and others) induce premature senescence of CD4+ T lymphocytes. Cocultivation with these bacteria led to the acquisition of a senescent phenotype (SASP) in T cells, with increased levels of cytokines IL-13, IL-17, IFN-γ, and markers of DNA damage (ATM, γH2A.X). Molecular analysis of senescence‑induction pathways revealed increased expression of phosphorylated p53 protein, accompanied by intense nuclear localization of p21CIP1/WAF1 (Mathiasen et al., 2021). It has previously been shown that the microbiota initiates neutrophil aging through activation of signaling pathways mediated by Toll‑like receptors TLR2 and TLR4, as well as the adaptor protein myeloid differentiation factor 88 (Myd88). Notably, senescent neutrophils do not express classical markers of cellular senescence but exhibit elevated CXCR4 levels, which contributes to their reduced numbers in the bone marrow. Consequently, neutrophil production is inhibited through signaling cascades involving IL‑17 and granulocyte colony‑stimulating factor (G‑CSF). Moreover, aging neutrophils are characterized by decreased L‑selectin expression. Functional analysis showed that these cells have impaired migratory capacity and attenuated pro‑inflammatory functions (Zhang et al., 2015).

In the context of B‑lymphocyte senescence, it has been demonstrated that this process is also induced by the bacterial microbiota, leading to reduced production of immunoglobulin A (IgA) in the intestine and, as a result, exerting a modulatory effect on the composition and functional state of the microbiota (Kawamoto et al., 2023).

## Bacteria as negative modulators of aging

A number of studies have demonstrated the positive influence of certain bacterial strains on processes associated with delayed aging. In 2025, L. Liu and colleagues investigated the effects of bacteria from plant roots on the lifespan of the nematode Caenorhabditis elegans. The most notable was the strain Mycobacterium sp. Root265, which synthesizes polysaccharides and arabinogalactan peptidoglycan, enhancing protein homeostasis and increasing lifespan through DAF-16-dependent and -independent pathways (Liu L. et al., 2025). Using the same nematode model, it was demonstrated that administration of live lactic acid bacteria, in particular Lactobacillus rhamnosus CNCM I-3690, increases their viability by 30 % and also extends the mean lifespan of the worms by 20 %. This effect is achieved through activation of DAF-16, which is associated with insulin-like signaling (Grompone et al., 2012).

Research using mouse models has shown that the bacterium Parabacteroides merdae can inhibit aging processes. It produces branched-chain amino acids that are converted into isovalerate, 2-methylbutyrate, and isobutyrate. In a mouse model of induced accelerated aging (under D-galactose administration), treatment with these metabolites reduced oxidative stress and inflammation, improved muscle function, increased acetylcholine levels in the brain, and normalized blood glucose levels (Azman, Zakaria, 2019). Microbiota analysis revealed an increase in beneficial bacteria, such as Bifidobacterium pseudolongum (Wang H. et al., 2025).

In a study conducted on healthy elderly individuals, it was found that administration of the probiotic BB68S – a specific strain of Bifidobacterium longum – significantly improved the cognitive functions of the participants. Specifically, improvements were observed in immediate memory, visuospatial and constructive memory, attention, as well as delayed memory. Furthermore, BB68S administration had a beneficial effect on the gut microbiota, promoting an increase in the relative abundance of beneficial bacteria such as Lachnospira, Bifidobacterium, Dorea, and Cellulosilyticum, while simultaneously reducing the abundance of bacteria associated with cognitive impairment, including Collinsella, Parabacteroides, Tyzzerella, Bilophila, and others (Shi et al., 2022).

Accumulated scientific evidence convincingly indicates that chronic and recurrent bacterial infections represent a significant factor contributing to the acceleration of systemic aging and the development of age-associated pathologies. The primary mechanism underlying this effect is the persistent activation of classical pro-inflammatory and cellular stress molecular pathways, the key ones of which are summarized in Figure 2a.

**Fig. 2.**
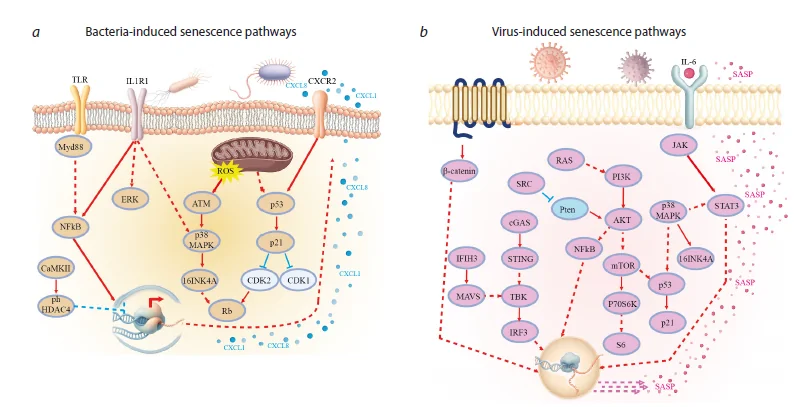
Illustration of molecular signaling pathways involved in senescence processes activated by (a) bacteria and (b) viruses. Solid arrows indicate the molecular connections discussed in this review, while dashed arrows represent established, known molecular connections.

Despite the current understanding of general mechanisms, the fundamental question of genetic predisposition to frequent or severe bacterial infections capable of inducing premature aging remains largely underexplored. For instance, although researchers have identified monogenic susceptibility variants for M. tuberculosis, data for the vast majority of other bacteria remain extremely fragmentary. Groundbreaking advances in microbiome research, especially the study of interactions between both commensal and pathogenic bacteria and host cells, are crucial for identifying novel molecular signaling pathways involved in lifespan regulation. These insights are essential for advancing this field further.

## Viruses as modulators of aging

In contrast to bacterial infections, viral infections exert an exclusively stimulatory effect on cellular senescence processes. Additionally, increasing evidence points to a genetic predisposition that worsens the course and consequences of viral infections. Viruses represent complex regulators of aging processes. Viruses act as complex regulators of cellular senescence: some trigger senescence as a protective measure to limit their replication, while others exploit senescent cells to facilitate their propagation. This interaction contributes to pathological conditions during viral infections, especially in elderly individuals, through mechanisms such as cytokine storms and immune response dysfunction (Seoane et al., 2020).

Although there is no evidence supporting a rejuvenating effect of viral infections, the role of viruses in inducing senescence remains unclear. Some studies report that certain viruses, such as herpesviruses, increase cellular senescence and accelerate tissue aging. However, other research either does not confirm these effects or finds them only in specific populations or after prolonged infection. These discrepancies may result from differences in research models (cell cultures, animal studies, or clinical data), experimental conditions, virus types, and individual genetic predispositions. Additionally, the impact of viruses may vary depending on the infection stage, the patient’s immune status, and comorbidities. This highlights the need for further research to clarify viruses’ exact role in aging and to identify potential therapeutic targets.

Recently, significant attention has focused on the SARSCoV- 2 virus. Studies indicate that it induces cellular senescence in infected cells as a stress response. In COVID-19 patients, senescence markers have been detected in the airways, alongside elevated levels of SASP factors in the blood. Senolytic drugs, such as navitoclax and the combination of dasatinib with quercetin, have demonstrated potential in eliminating virus-induced senescent cells and reducing lung damage in animal models (Lee et al., 2021). Additionally, the viral spike protein accelerates cellular senescence by increasing Cdc42 expression, which activates the Wnt/β-catenin pathway. Treatment with Cdc42 inhibitors has been shown to reduce lung damage and inflammation (Nong et al., 2024).

Genetics also plays a significant role in the risk of SARS-CoV-2 infection. Specific genetic markers asso- ciated with increased susceptibility have been identified, including variants rs11246068-CC, rs12252-CC, rs1990760, and rs2111485 in the IFITM3 gene; rs5742933- GG in the ORMDL1 gene; rs35337543-CG in the IFIH1 gene; and the GGGCT haplotype, which includes polymorphisms rs2070788, rs2298659, rs17854725, rs12329760, and rs3787950 in the TMPRSS2 gene. These genes are directly involved in antiviral immune responses and viral replication. These genetic variants are important markers of infection susceptibility and may also play a role in accelerating virus-induced aging processes (Beckmann, Becker, 2021; Xu et al., 2022; Majeed et al., 2024; Azcarate et al., 2025).

Similarly, infection with influenza A virus (IAV) leads to the development of both acute and chronic lung tissue damage. In a mouse study using a sublethal dose of the H1N1p2009 virus, cellular senescence was detected, marked by increased expression of p16, p21, β-galactosidase, and the DNA damage marker γ-H2A.X in lung tissue. The process of cellular senescence began as early as day four post-infection in the bronchial epithelium and subsequently spread to the lung parenchyma. Thus, virus-induced cellular senescence contributes to the development of influenza-related pulmonary complications (Lipskaia et al., 2025). Genetic factors also affect susceptibility and severity of influenza A infections. Analysis of patients with severe pneumonia identified polymorphisms on chromosomes 1 and 17 that may influence risk, including variants in FCGR2A (rs1801274), RPAIN (rs8070740), and C1QBP (rs3786054) genes (Zúñiga et al., 2012). Additional studies found polymorphisms in IFITM3 (rs12252), and Toll-like receptor genes TLR2, TLR3, and TLR4 (rs5743708, rs5743313, rs5743315, rs4986790, and rs4986791) (Hidaka et al., 2006; Esposito et al., 2012; Chen C. et al., 2016). Furthermore, polymorphisms in CD55 (rs2564978) and C1QBP (rs3786054) have been linked to increased risk of fatal outcomes of influenza infection (Chatzopoulou et al., 2019).

Human respiratory syncytial virus (HRSV) is a leading cause of lower respiratory tract infections. Infection with HRSV induces cell cycle arrest at the G1 or G2/M phases, depending on the cell type, through accumulation of proteins p53 and p21 (Bian et al., 2012). It also triggers the expression of DNA damage markers and proliferation arrest markers, such as phosphorylated ATM (ph-ATM) and γH2A.X (Martínez et al., 2016). Regarding genetic susceptibility, a single nucleotide polymorphism in the olfactory receptor gene OR13C5, rs199665292, has been identified as a promising candidate variant. Additionally, variants in the major histocompatibility complex HLA genes (HLA-DQA1, HLA-DPB1) and the mucin 4 gene (MUC4) have been implicated (Salas et al., 2017). Mutations in TLR4, specifically Asp299Gly and Thr399Ile, either alone or combined, occur more frequently in patients with severe disease forms (Tulic et al., 2007).

Human cytomegalovirus (HCMV) is the causative agent of cytomegaly. To replicate its double-stranded DNA genome, HCMV induces significant alterations in cellular homeostasis, analogous to cellular senescence. It promotes decreased expression of Lamin B1 and KI67, alongside increased secretion of pro-inflammatory cytokines IL-6 and IL-8 in various cell types, which are well-established markers of cellular senescence (Raviola et al., 2024). Supporting this, T. Hasegawa et al. (2023) found HCMV DNA and RNA in normal human skin, with viral levels significantly higher in samples from elderly individuals, suggesting enhanced viral replication in senescent cells (Hasegawa et al., 2023). While age-related predisposition to HCMV infection is recognized, several genetic variants have been proposed as contributors to infection progression, though their clinical relevance remains uncertain. The rs11686168 polymorphism in the CDC42EP3 gene is the most promising candidate, although its functional role is not yet understood (Casto et al., 2021). It is hypothesized that CDC42EP3 may not directly influence the virus but could affect cellular processes that viruses exploit.

Human immunodeficiency virus type 1 (HIV-1), belonging to the retrovirus family, promotes premature aging in patients with chronic infection. Individuals with HIV infection exhibit decreased bone mineral density, as well as an increased prevalence of osteopenia and osteoporosis. This phenomenon is attributed to the viral proteins Tat and Nef, which induce premature aging of bone marrow mesenchymal stem cells, reducing their proliferation due to oxidative stress and mitochondrial dysfunction. Consequently, their ability to differentiate into osteoblasts is diminished. The Tat protein additionally activates NF-κB and stimulates the secretion of pro-inflammatory cytokines, contributing to the inflammatory response (Beaupere et al., 2015). HIV-1 also induces senescence in microglial cells, characterized by increased β-galactosidase activity, elevated p21, and higher levels of cytokines IL-6 and IL-8, alongside decreased expression of SIRT3, a mitochondrial enzyme essential for cellular homeostasis and metabolism. These changes coincide with increased mitochondrial oxidative stress and the development of a SASP (Chen N.C. et al., 2018; Thangaraj et al., 2021).

In addition to viral infections that accelerate aging (Lee et al., 2021), the host genome contains retroelements – highly mobile DNA sequences integrated throughout the genome, many of which also exist as extrachromosomal DNA. Reactivation of endogenous retroviruses (ERVs), a subtype of these retroelements, has been linked to the aging process and is driven by an epigenetic switch. A class of ERVs is epigenetically silenced in proliferating cells but becomes activated during replicative senescence, RAS- and Akt-induced senescence, or after HDAC4 gene knockout (Di Giorgio et al., 2024). Moreover, ERVs are activated by the transcription factor ATF3, leading to the formation of double-stranded RNAs that trigger the RIGI/ MDA5-MAVS signaling pathway and stimulate type I interferon (IFN-I) production in senescent fibroblasts. This aging-associated molecular mechanism has been observed in tissues from healthy elderly individuals as well as patients with Hutchinson–Gilford progeria syndrome (Mao et al., 2024).

HIV-1 is among the most extensively studied viruses, with recent research showing that susceptibility to HIV-1 acquisition is a polygenic and heritable trait. Heritability estimates based on single nucleotide polymorphisms (SNPs) account for 28 to 42 % of the variation in susceptibility across the population. Genetic analyses have identified EFCAB14 as a novel gene linked to HIV-1 susceptibility. Additionally, a higher genetic risk for HIV-1 acquisition correlates with lower levels of the chemokine ligand CCL17 (Powell et al., 2020). It is known that HIV-1 uses the CCR5 co-receptor for infection, and in some cases, the CCR2 and CXCR4 co-receptors. Consequently, genetic mutations in these receptors represent an important factor influencing susceptibility to infection. The frequency of heterozygous mutations in the CCR5 gene is significantly elevated among individuals who are able to tolerate infection for many years. Moreover, the CCR5delta32 and CCR2-64I mutations may act as factors limiting HIV-1 infection and also confer a dominant phenotype of delayed disease progression in infected patients (Dean et al., 1996; Smith et al., 1997; Martin, 1998).

Thus, numerous studies confirm that viruses act as potent inducers of cellular senescence. They initiate this process through various molecular pathways, as schematically represented in Figure 2b. It is known that individual susceptibility to viral infections and the severity of their course have a genetic basis. However, most of the identified predisposition genes encode proteins directly involved in virus recognition and the formation of innate or adaptive immune responses (e. g. interferon pathway genes, pattern recognition receptors) (Table S1 in Supplementary Material)1. However, functional studies directly linking these genetic variants to the modulation of virus-induced cellular senescence are currently lacking. Consequently, a critical question remains: does genetic predisposition influence the intensity of the senescent response to infection and what are the underlying molecular mechanisms? Addressing this could open promising avenues for targeted control and therapeutic regulation of virus-induced cellular senescence.

## Conclusion

This review summarizes data on various pathogenic modulators that can both positively and negatively influence aging processes. It provides a comprehensive analysis of how pathogenic agents affect classical molecular senescence pathways and trigger novel cascades leading to age-related changes. The review also highlights candidate genes that contribute to genetic predisposition to infections, which in turn can accelerate or delay aging processes (Table S1). Studies conducted on model organisms and cell cultures demonstrate that exposure to even a single such factor can induce irreversible structural and functional changes. Investigating the molecular mechanisms of pathogeninduced senescence is a vital interdisciplinary field that integrates infectious disease research, gerontology, genetics, and the study of age-related diseases. Understanding these mechanisms is essential not only for deepening our knowledge of aging but also for opening new avenues in the prevention and treatment of age-related conditions, as well as for promoting healthy lifespan extension. This approach frames aging as the combined outcome of external factors, such as infections, and internal biological processes. It lays the groundwork for a new generation of preventive medicine focused not on treating individual diseases but on systemically slowing aging by addressing its root causes, including pathogenic influences. Such a strategy has the potential to prolong the active, healthy years of life, lessen the impact of age-related diseases, and enhance quality of life for older adults.

The epidemiological significance of the bacteria discussed is extremely high: for example, H. pylori infects approximately half of the world’s population, with the highest prevalence in low-income countries (Li et al., 2023). Representatives of periodontopathogens, such as P. gingivalis, are widespread among the adult population, thus linking chronic infections with an increased risk of age-associated diseases (Mei et al., 2020). The key conclusion concerns the dualistic role of bacteria. On the one hand, certain pathogens can act as inducers of aging through the activation of SASP in cells, cell cycle arrest, and immunosenescence. On the other hand, commensal bacteria, such as P. merdae, exhibit geroprotective potential, counteracting oxidative stress and inflammation through the production of specific metabolites. So, aging can be viewed not only as an internal biological process but also as a phenomenon influenced by the human microbiota. Aging processes are driven by the interplay of cell-autonomous and non-cell-autonomous mechanisms. In particular, senescence induction caused by bacterial infection is primarily regulated by classical molecular pathways such as Erk, p53-p21, and ATM-p38. Furthermore, additional signaling cascades have been identified, mediated through the CXCR2, IL-1R1, CaMKII-HDAC4, and TLR-MyD88 mechanisms. It should be noted that activation of the CXCR2 signaling pathway has been demonstrated in the context of oncological aging, whereas the TLR-MyD88 pathway is associated with immune aging. At the same time, the mechanisms of so-called anti-aging induced by probiotics remain, in most cases, insufficiently understood and require further investigation.

Unlike bacteria, viral infections have an exclusively negative impact on cellular senescence, with no evidence to date of any rejuvenating effects. Despite this overall detrimental influence, viruses regulate aging processes through diverse molecular pathways, including PI3K– AKT–p70S6K, p38MAPK, SRC, Wnt/β-catenin, p53, IL-6-JAK-STAT3, and NF-κB. Retroviruses specifically engage signaling cascades such as IRF3, cGAS–STING, RAS, IFIH1-MAVS, and ATF3–RIG-I/MDA5–MAVS. Understanding these virus-induced aging mechanisms is critically important for developing therapeutic strategies to treat viral infections and promote healthy aging. However, research in this area remains limited, and there is a pressing need to identify therapeutic targets and develop effective pharmaceutical interventions.

Two main types of cellular senescence are recognized: senescence of immune cells (immunosenescence) and senescence of non-immune cells. Growing evidence shows that senescent non-immune cells, especially tumor cells, can induce senescence in immune cells. For example, senescent cancer cells increase expression of programmed death-ligand 1 (PD-L1) on their surface or release extracellular vesicles containing PD-L1, which trigger T-lymphocyte senescence (Majewska et al., 2024; Hwang et al., 2025; Ma et al., 2025). Conversely, immune cell senescence exerts systemic effects on the organism (Liu Z. et al., 2023). Notably, senescent cells within a single population exhibit significant heterogeneity, complicating the development of effective senolytic agents targeting these cells. Current approaches leverage machine learning for subclassification based on morphological features alongside omics technologies for molecular profiling (Kamat et al., 2025; Wang Z. et al., 2025). However, existing data remain insufficient to define a unique senescence phenotype. It remains unresolved whether the observed heterogeneity reflects distinct subpopulations or temporal phenotypic evolution within the same cell population (Bitencourt et al., 2024).

It is important to recognize that research on senescence mechanisms often faces certain biases, as it tends to focus primarily on well-established signaling pathways and markers, potentially overlooking many significant but less studied mechanisms. Additionally, there is a methodological challenge stemming from discrepancies between in vitro and in vivo study results. Model systems frequently fail to fully replicate the complexity of human tissues and whole organisms, limiting the applicability of findings. The heterogeneity of senescent cells, in morphology and function, also complicates their accurate identification and evaluation within tissues. These limitations must be carefully considered when interpreting results and it is important to highlight the need for further research to improve the translational relevance of findings and to develop effective preventive strategies.

Understanding the molecular mechanisms of pathogeninduced senescence holds great potential for developing novel diagnostic tools that enable early detection of infection- related pathological changes and timely preventive interventions. Moreover, elucidating the role of pathogens in driving aging paves the way for therapeutic strategies aimed at mitigating inflammatory responses and delaying aging processes in patients with chronic or prolonged infections. Incorporating these approaches into clinical practice could significantly enhance the quality of life for elderly individuals, lower the risk of age-related diseases, and broaden the scope of comprehensive management for age-associated conditions. Therefore, this research area not only advances academic knowledge but also lays the groundwork for future medical breakthroughs and the extension of a healthy human lifespan.

## Conflict of interest

The authors declare no conflict of interest.
